# Intelligent imaging triage systems for reducing waiting anxiety: a narrative review

**DOI:** 10.3389/fpsyt.2026.1716109

**Published:** 2026-02-04

**Authors:** Qin Zhao, Haiyu Wang, Qingfeng Li

**Affiliations:** Department of Medical Imaging, Dazhou Dachuan District People’s Hospital, Dazhou Third People’s Hospital, Dazhou, Sichuan, China

**Keywords:** artificial intelligence intervention, intelligent imaging triage system, medical imaging, optimization of medical workflow, waiting anxiety

## Abstract

The delay in medical imaging exams can cause significant anxiety, impacting patient adherence, imaging quality, and overall experience. Intelligent imaging triage systems, driven by artificial intelligence in radiology, aim to improve examination processes and reduce patient anxiety. This review discusses the prevalence of patient anxiety during waiting periods, as well as the physiological and psychological mechanisms. In addition, the structure and functions of these systems, their current use in top domestic hospitals and international healthcare systems, and initial findings on anxiety reduction and enhanced patient satisfaction are analyzed. Some methods were proposed to address the challenges, such as limited evidence, sample representation, and durability assessment. Potential technological advancements, innovations in clinical services, and future interdisciplinary opportunities and policy implications were explored. Intelligent imaging triage systems have the potential to improve the medical workflow efficiency and provide emotional support within patient-centered care. This review concludes that while promising, the widespread adoption of these systems necessitates more robust evidence, interdisciplinary collaboration, and supportive policies.

## 1 Introduction

Medical imaging exams are becoming increasingly important in disease diagnosis and management. Concurrently, “waiting anxiety” has had a significant impact on patients’ mental health and diagnostic reasoning worldwide. 52.5% of patients undergoing magnetic resonance imaging (MRI) experience notable anxiety beforehand, which rises to 66.5% post-exam, highlighting that even the relatively brief waiting time contributes significantly to exam-related anxiety (1). In an Italian single-center prospective study, 29% of MRI patients reported high anxiety prior to the exam, often unrelated to cancer diagnosis but rather to the uncertainties of the procedure (2). Data from a broader multi-site survey in western Saudi Arabia showed that 55.9% of MRI patients experienced moderate anxiety during the perceived waiting period, 51% felt panic, and 74% desired clearer surgical information to alleviate anxiety (3). Anxiety has significant implications for clinical outcomes and healthcare services, potentially hindering patient understanding of treatment plans and adherence to follow-up care, resulting in inevitable complications (4). Anxiety-driven repeat imaging across departments undermines diagnostic accuracy and increases inefficiencies in imaging protocols (5). Reduced patient compliance due to heightened anxiety can degrade image quality, necessitating repeat imaging and escalating healthcare costs. Artificial Intelligence (AI)-based imaging triage systems are currently being piloted in hospitals, are moving toward standardization, and thus offers a viable solution. These systems can dynamically analyze exam requisitions, imaging equipment, bookings, and patient data, enabling automated triage, sequencing, and communication management. For example, studies on AI in tuberculosis screening demonstrated that AI triage systems could maintain physician productivity and high patient satisfaction, indicating their potential to streamline radiology processes (6). The integration of AI with imaging processes has been shown to decrease report wait times, and potentially ease patient anxiety associated with awaiting a diagnosis (7). Although they are effective in reducing anxiety related to uncertainty about patient care requests and waiting times (8), the deployment of intelligent triage systems remains limited. Current studies on “waiting anxiety” in medical imaging often focus on phenomenological aspects, with interventions mostly unstructured and rarely AI-driven. However, AI-enhanced imaging triage systems, including advanced scheduling, patient communication, and feedback mechanisms, are not merely enhancements to the radiographic process. They offer significant opportunities to alleviate anxiety at the system level and represent a key focus for the future development of intelligent healthcare systems.

This narrative review therefore aims to synthesize current evidence on the efficacy of AI-powered imaging triage systems in mitigating waiting anxiety, evaluate their technological and clinical implementation, and identify key challenges and future directions for research and policy. This work aimed to contribute to multidisciplinary research and policy development. By synthesizing evidence and offering strategic insights, we seek to transform intelligent triage systems from mere technological tools into integrated solutions that include psychological support, thereby promoting a more compassionate and effective medical imaging service.

## 2 Clinical context and influencing factors of waiting anxiety in patients

### 2.1 Current clinical context of waiting anxiety in medical imaging examination

**Figure 1:**
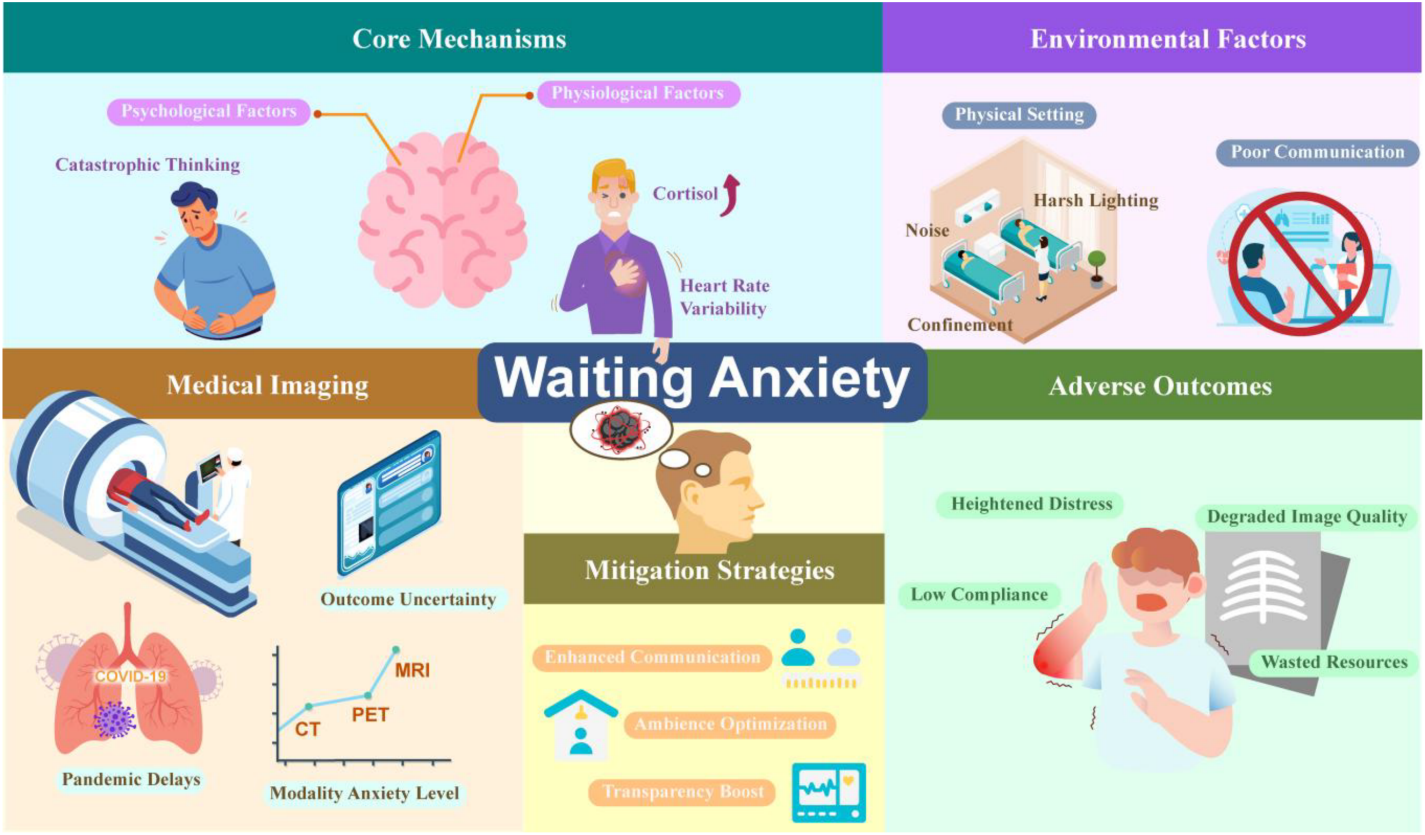
Clinical context and influencing factors of waiting anxiety.

**Figure 1** summarizes the core mechanisms, environmental contributors, and clinical consequences of waiting anxiety in medical imaging examinations. Psychological factors (e.g., catastrophic thinking and uncertainty) and physiological stress responses (e.g., elevated cortisol and reduced heart rate variability) interact to generate waiting anxiety. External stressors, including confined physical settings, noise, harsh lighting, and poor communication, further exacerbate this condition. Imaging-related factors such as modality-specific anxiety (MRI > PET/CT > CT), outcome uncertainty, and pandemic-related delays contribute to its development. Unresolved waiting anxiety may result in heightened distress, reduced patient compliance, degraded image quality, and inefficient use of medical resources. The figure also highlights key mitigation strategies, including enhanced communication, transparency, and environmental optimization, which form the conceptual basis for AI-enabled imaging triage interventions.

Medical imaging examinations are a critical component of clinical decision-making, yet the growing evidence suggests that patients often experience considerable anxiety while awaiting these procedures or their results (**Figure 1**). This psychological phenomenon, commonly referred to as “waiting anxiety,” has been extensively documented worldwide. A cross-sectional prospective study of 488 outpatients by Zewar et al. found that approximately 49% of patients undergoing CT, MRI, or ultrasound examinations exhibited significant levels of anxiety, with 48% reporting that their anxiety was closely tied to the uncertainty of their results (9). Similarly, an Australian study of 777 patients found that 51% experienced anxiety after their imaging examinations, with 36% reporting highly persistent anxiety throughout the entire imaging process (10). It has been reported that the prevalence of anxiety among patients undergoing PET/CT examinations exceeds 60%. However, enhancing pre-procedure communication effectively reduced average anxiety levels, with all patients reporting average anxiety levels below 30% (11). These findings underscore the substantial impact of “waiting anxiety” on individuals undergoing medical imaging procedures and emphasize the need for healthcare providers to address this issue and implement strategies to alleviate patient anxiety.

Imaging modalities exhibit systematic differences in terms of intensity measurement, traditionally ranked as MRI > PET/CT > CT. A recent systematic review reported that MRI examinations induced more anxiety in patients, attributed to longer waiting times, confined seating, and high noise levels that hinder concentration on reassurance messages (12). Similarly, a study comparing distress levels in PET/MRI and PET/CT patients found that despite the challenges of MRI imaging, it caused less psychological distress than CT scans, with CT scans recording distress levels exceeding 40 for first-time examinees (13).

Since the onset of the COVID-19 pandemic, anxiety related to imaging schedules, protocols, and altered care pathways has surged. Hospitals are perceived as high-risk areas for infection, leading to delays in scheduling, reporting, and requests, which exacerbate patients’ anxiety about missing imaging appointments. The uncertainty surrounding multifactorial and unknown infection risks faced by patients awaiting evaluation further intensifies this anxiety. Previously, such concerns were often overlooked, particularly in contexts like PET/CT exams with coronary computed tomography angiography (CTA) (14). These issues warrant serious investigation to cultivate faster and more robust mechanical insights in future research.

### 2.2 Mechanisms underlying waiting anxiety

Waiting anxiety stems from psychological and physiological factors rather than occurring randomly. Uncertainty is a well-known anxiety trigger, proven to exacerbate anxiety through laboratory waiting paradigms. Ambiguous outcomes amplify perceived threats and loss of control, which intensifies anxiety (8). Fear-driven unpredictable events activate prefrontal cortex and amygdala pathways, fostering catastrophic thinking (8). The lack of transparency in waiting for imaging exams diminishes patients’ sense of control, whereas enhancing control can alleviate anxiety. Physiologically, waiting anxiety can activate the sympathetic nervous system, causing various changes. A 2024 study reported elevated salivary cortisol and reduced heart rate variability in highly anxious patients before MRI exams (15), indicating autonomic imbalance. These physiological impairments manifest as increased pain, difficulty breathing, and muscle tension, as well as reduced cooperation during imaging, which may affect image quality and examination efficiency. Anxious patients also experience higher re-scan rates and waste medical resources.

### 2.3 Environmental and service factors related to waiting anxiety

In addition to personal factors, environmental conditions and inadequate service provision play a crucial role in exacerbating waiting anxiety. Traditional imaging environments are characterized by noise, enclosed spaces, and bright lights, which exacerbate this anxiety. Nuclear medicine research has shown that soft lighting and soothing music can significantly reduce this anxiety (16). Studies have shown that there is widespread uncertainty regarding imaging procedures, priorities, and results timelines. Nearly half of outpatients reported insufficient communication about examination details and waiting times, leading to confusion (17). Enhancing transparency, patient experience, and communication is critical for intervention measures and should be incorporated into future smart healthcare systems.

## 3 Technical principles and current development of AI-based imaging triage systems

### 3.1 Concept, structure, and classification

**Figure 2:**
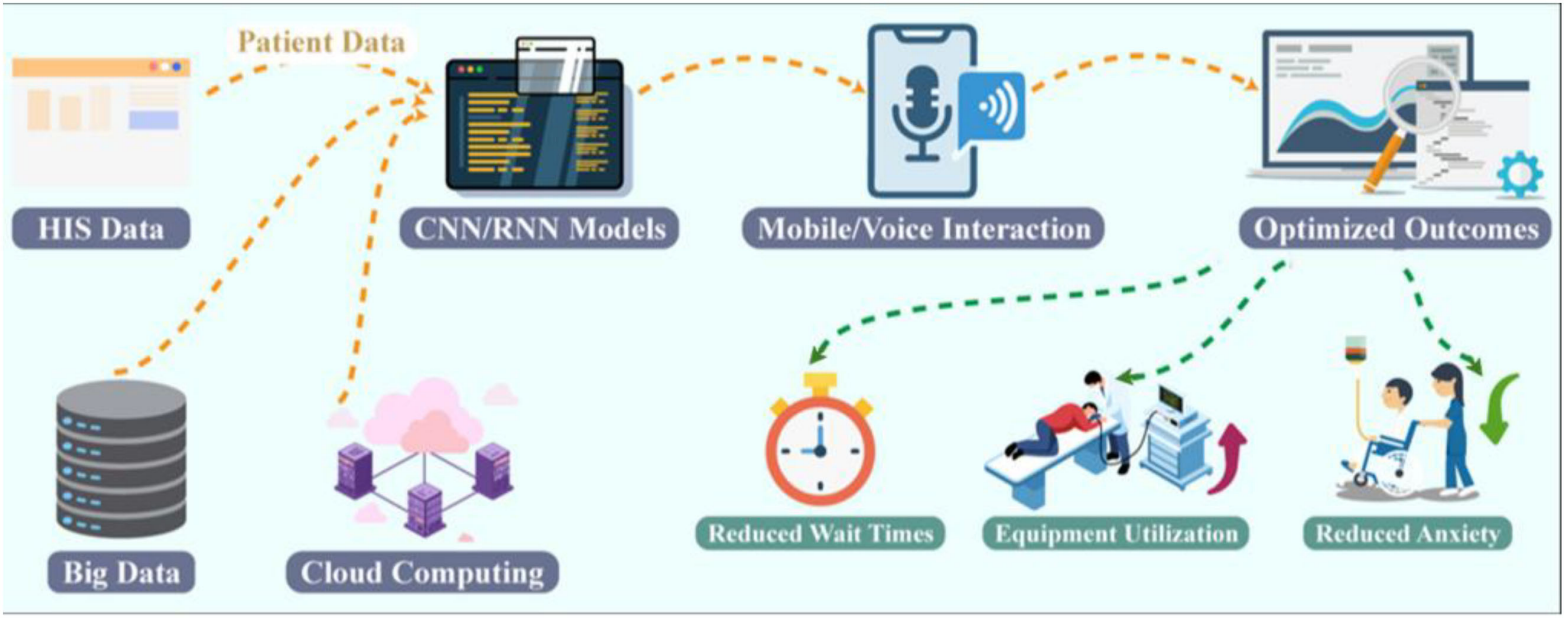
Technical principles and current development of AI-based imaging triage systems. Patient and hospital information system (HIS) data are integrated with big data and cloud computing infrastructures and processed by intelligent algorithmic layers, including CNN- and RNN-based models, to support imaging prioritization and scheduling. The human–computer interaction layer enables real-time feedback through mobile applications and voice-based interfaces. This closed-loop framework optimizes imaging workflows by reducing waiting times, improving equipment utilization, and alleviating patient anxiety.

As the demand for medical imaging increases and examinations become more complex, traditional triage methods—relying on manual queuing and strict scheduling—fail to meet modern healthcare standards for efficiency and patient experience (**Figure 2**). To address these challenges, AI-based Imaging Triage Systems have been developed. These systems employ artificial intelligence to prioritize, schedule, and provide feedback on imaging requests, thereby optimizing workflow and individual management. Benefits include enhanced capacity utilization of imaging equipment, reduced wait times, and decreased patient anxiety through more timely feedback. Consequently, AI Imaging Triage Systems are increasingly integrated into intelligent imaging service frameworks (18). AI-based imaging triage system consists of three main components: the sensing/input layer, the intelligent algorithmic layer, and the human-computer interaction (HCI) interface. The sensing/input layer gathers data from hospital information systems, including patient demographics, chief complaints, and imaging requests. The intelligent algorithm layer uses deep learning models and rule-based engines to predict timelines, prioritize requests, and optimize resource allocation (19). HCI layer enhances patient engagement through dynamic communication channels, such as mobile apps, web interfaces, and voice assistance. This closed-loop system is designed to provide real-time updates on queue status and waiting times, thereby enhancing awareness, engagement, and feedback.

### 3.2 Technological utilities and maturity

The growing demand for hospital imaging services necessitates a coordinated expansion of specific technological components to support the development of next-generation AI-driven imaging triage systems. Three key technological advances may drive the rapid development of AI imaging systems: deep learning, big data and cloud computing, and human-computer interaction design. AI modeling approaches, such as convolutional neural networks (CNNs) and recurrent neural networks (RNNs), offer robust and data-driven methods for modeling imaging appointment patterns and prioritizing schedules. For instance, a hybrid CNN-RNN model can classify patient complaints in relation to imaging needs with greater accuracy than standard rule-based systems, while maintaining a clinically relevant mean absolute error (MAE) of approximately five minutes for scheduling (19). Similarly, an end-to-end AI framework for stroke patients can autonomously segment cerebral vascular organization, quantify collateral circulation, and generate interoperable data to perform the entire workflow from imaging input to emergency triage recommendations (20). The rapid advancements in artificial intelligence (AI) algorithms have expanded their capabilities beyond mere image recognition, enabling their integration into complex decision-making processes, such as triage systems. The increasing reliance on cloud computing is critical for the anticipated growth in real-time computing and inter-system communication required by hospital intelligent triage systems (21). Iftikhar et al. (2022) recently described a task scheduling framework leveraging convolutional neural networks (CNNs) in a cloud-edge collaborative environment (22). The results demonstrated promising energy efficiency when operating under high-volume imaging scheduling requests with characteristics of extreme tail latency. Specifically, the task consumed 17% less energy while exhibiting a 10.4% higher effectiveness for task loads exceeding 105 concurrent requests. These findings suggest that the field has progressed to the point where a shift from localized implementations towards a more platform-based approach is now feasible, and leverages an imaging-as-a-service model aligned with the cloud-commerce paradigm.

### 3.3 Developmental stages of AI-based imaging triage systems

AI-based imaging triage systems have evolved rapidly from early exploratory implementations to increasingly mature clinical applications. In the initial developmental stage, these systems primarily relied on rule-based heuristic methods, with scheduling logic manually designed by clinical or administrative staff. While functional, such approaches lacked adaptability and were insufficient for managing high-volume imaging demands or complex clinical scenarios.

The subsequent growth stage was driven by advances in artificial intelligence, particularly deep learning techniques such as convolutional neural networks (CNNs) and recurrent neural networks (RNNs). These data-driven models enabled automated prioritization, dynamic urgency assessment, and more accurate prediction of waiting times, significantly improving workflow efficiency and responsiveness. During this phase, imaging triage systems transitioned from static scheduling tools to adaptive decision-support platforms capable of learning from real-world clinical data.

More recently, AI-based imaging triage systems have entered a maturation stage, characterized by integration with hospital information systems and other clinical platforms. Emerging evidence suggests that such systems can enhance the management and flow of patient information within complex digital healthcare ecosystems, potentially improving interoperability and coordination across departments and institutions (23). Furthermore, AI-driven tools that support patient engagement, such as automated appointment reminders and real-time feedback mechanisms, may contribute to increased patient autonomy and satisfaction, reinforcing the human-centered dimension of imaging services (24).

### 3.4 Future trends of AI-based imaging triage system

Several key trends are expected to shape the future development of AI-based imaging triage systems. Firstly, these systems are likely to evolve into precision-oriented care tools by integrating multimodal data, including clinical diagnoses, imaging requirements, emergency status, and patient-specific characteristics, to generate individualized scheduling and prioritization strategies.

Secondly, AI imaging triage systems are expected to become more pervasive, transitioning from isolated hospital-based applications to regional or network-level platforms. Such interconnected systems would enable coordinated imaging resource allocation, appointment scheduling, and result sharing across healthcare facilities, thereby improving continuity of care and system-wide efficiency.

Thirdly, ethical, legal, and regulatory frameworks will play an increasingly central role in guiding system design and deployment. Strengthened governance related to data privacy, algorithmic transparency, and accountability will be essential to ensure safe, equitable, and trustworthy integration of AI triage technologies into routine clinical practice.

Overall, AI-based imaging triage is transitioning from a tool primarily aimed at improving operational efficiency to a comprehensive intelligent system that integrates advanced imaging technologies with patient-centered healthcare services. This evolution is expected to drive innovation in outpatient and departmental digital healthcare, positively influencing patient experience, healthcare equity, and the long-term sustainability of healthcare systems.

## 4 Current application of intelligent imaging triage systems in patient anxiety intervention during waiting periods

### 4.1 Current clinical application status domestically and internationally

**Figure 3:**
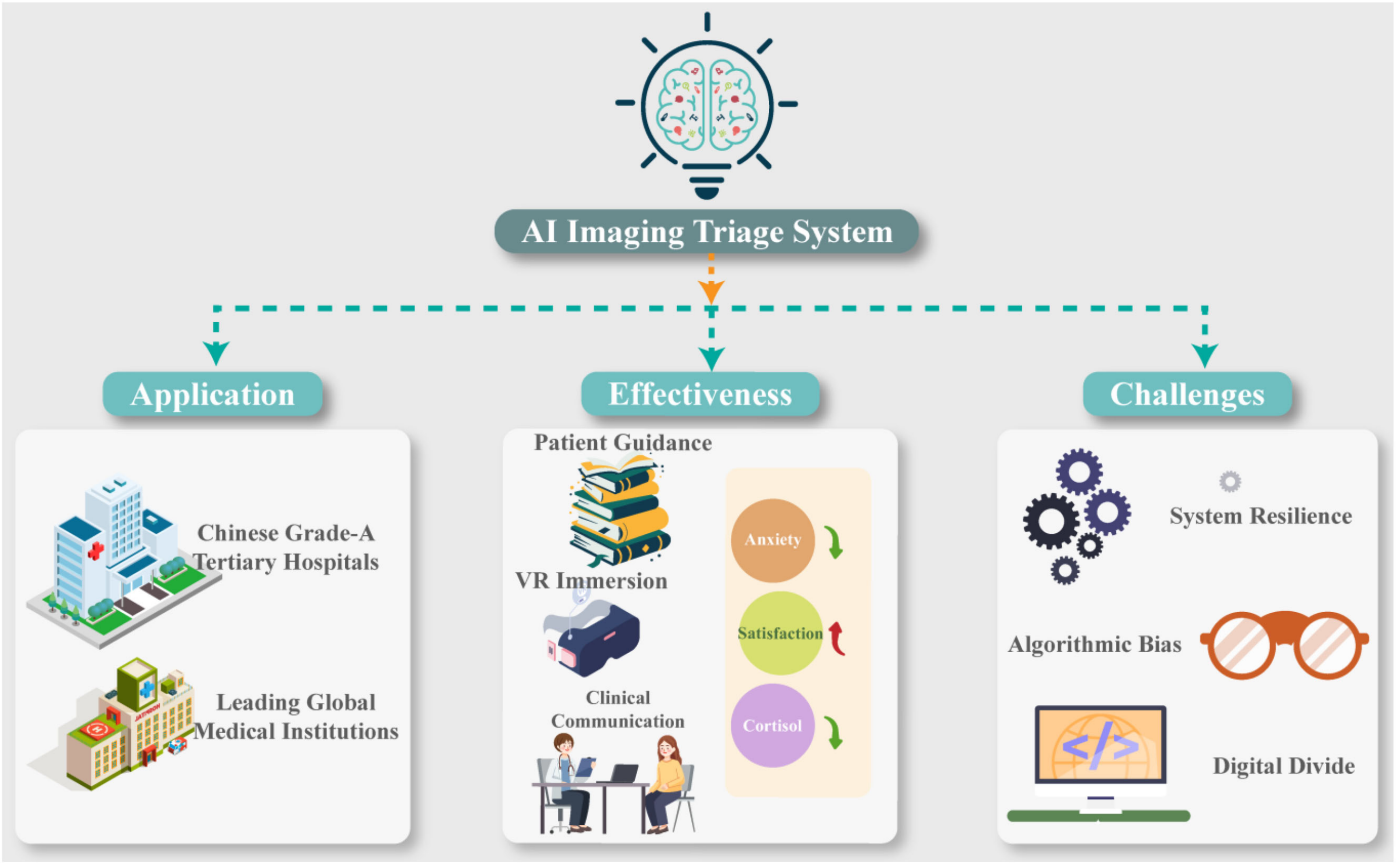
Applications, effectiveness and challenges of AI imaging triage system. Current applications include large tertiary hospitals in China and leading international medical institutions. Reported effectiveness involves improved patient guidance, immersive interventions, enhanced clinical communication, reduced anxiety, increased satisfaction, and favorable physiological stress responses. Major challenges include system resilience, algorithmic bias, and the digital divide, particularly among elderly populations. All elements are original illustrations created by the authors.

In recent years, driven by the rapid development of intelligent medical policies and artificial intelligence imaging technology, intelligent imaging triage systems have been increasingly adopted by major medical institutions (**Figure 3**). In China, prominent hospitals, such as Huashan Hospital, Zhongshan Hospital affiliated with Fudan University, and the First Affiliated Hospital of Sun Yat-sen University, implement AI scheduling systems in high-demand areas like MRI and CT rooms. These systems can perform real-time analysis and queuing strategies based on patient appointment availability and complaints, and communicate the results to patients via mini-applications.

Mayo Clinic and its Center for AI in Imaging (CAII) are global leaders in AI-driven research and clinical applications. CAII has developed an AI platform for managing cardiology CT and MRI procedures, workflow informatics, and emergency CTA. These systems employ multimodal feedback to enhance collaboration among doctors, technologists, and patients. A Mayo Clinic study indicates that successful AI health system implementation typically requires an index period of 10–20 months for data standardization, algorithm deployment, and staff training (25). Once completing medical staff education is complete, these systems significantly improve operational efficiency, particularly in pre-examination notifications and reducing examination omission rates, benefiting high-volume clinical settings such as acute stroke and cardiovascular imaging scenarios.

In 2023, the UK’s National Health Service (NHS) further committed to integrating AI into medical imaging and diagnostics. The UK Department of Health allocated an additional £21 million to support NHS Trusts in implementing AI-based imaging triage and decision support systems. This funding aims to achieve nationwide coverage for stroke care networks and certain preliminary chest X-ray screening services to lower patient wait times while improving diagnostic efficiency (26). The integration of AI-enabled triage tools into remote collaboration platforms, such as the Mayo Clinic Telestroke Network, has demonstrated significant benefits in the management of acute stroke patients. These systems automatically analyze CT angiography images to detect large vessel occlusions (LVO) and transmit notifications through pre-established protocols without direct clinician input. This has significantly reduced the average time from initial patient transfer from a remote hospital to the receiving hospital where neurointerventional surgery begins by approximately 15 minutes. Additionally, the use of these AI-based technologies has greatly enhanced physicians’ confidence in diagnostic decision-making (27). National policy directives emphasize the importance of evaluating the utility of AI-based technologies in terms of enhancing patient experience and reducing unnecessary resource utilization, while also assessing their cost-effectiveness at the national level. The implementation of these AI-based triage tools on remote collaboration platforms demonstrates the potential of these technologies to improve the efficiency and effectiveness of healthcare services, particularly in acute stroke management. The integration of AI in medical imaging extends benefits beyond enhanced diagnostic accuracy. A thorough review of prominent institutions, such as GE, Siemens, Mayo Clinic, and Massachusetts General Hospital, reveals the positive effects of AI-driven imaging triage systems. These systems improve equipment utilization and reduce patient wait times between arrival and examination (28). Consequently, imaging technologies are evolving from a peripheral role to a central element of next-generation healthcare platforms (29).

International frameworks focus on integrated assessments of systematic improvements derived from process efficiencies, emotional outcomes, and service satisfaction, while considering ethical, patient safety, and equity issues in evidence-based discussions (30). Consequently, intelligent imaging triage systems are crucial in advancing patient-centered human-machine collaboration in radiology departments.

### 4.2 Evaluative research on anxiety intervention effectiveness

In recent randomized controlled trials (RCT), the impact of intelligent imaging triage systems on patient anxiety have been explored through State–Trait Anxiety Inventory (STAI) scores, physiological stress measures, and patient satisfaction scores as metrics. Yahagi et al. conducted RCT with 100 surgical patients, and compared ChatGPT-assisted nursing with nurse-guided nursing. The study found that the AI group had significantly lower STAI scores, improved information clarity, and greater confidence in decision-making during preoperative consultations, highlighting the value of AI systems in shaping patient expectations (31). In a randomized controlled trial, Bolejko and Hagell assessed MRI-related anxiety by self-report measures. Patients in the intervention group received structured informational brochures before imaging, which resulted in significantly lower rates of high anxiety (State-Trait Anxiety Inventory score > 55) compared to controls (odds ratio = 2.64). Additionally, the intervention group reported much higher satisfaction scores (32). Shimokawa et al. conducted a study in Japan utilizing audiovisual (AV) systems described as “patient-friendly audiovisual environments” for individuals undergoing MRI examinations. The authors reported that their intervention effectively lowered the anxiety in 81.8% of patients with self-reported high anxiety. There was an improvement compared to the standard care outcomes (42.9%), confirming the efficacy of immersive interactive designs in mitigating psychological stress during imaging procedures (33). The use of physiological indicators has been incorporated into the imaging field to record the efficacy of interventions. In a study using MRI examination information and communication interventions, Tazegul et al. reported that saliva cortisol levels decreased by 6% in the intervention group, while they increased by 18% in the control group (34). Similarly, Liu et al. found that the use of virtual reality (VR) technologies in their anxiety intervention led to significantly improved preoperative STAI scores, as well as increased self-efficacy and patient satisfaction in the VR group compared to controls (34). Furthermore, Dias et al. examined the nurse-led preoperative communication intervention and observed statistically significant decreases in STAI scores, although the differences in satisfaction scores were not statistically significant (35). Collectively, these findings demonstrate the effectiveness of intelligent, informational, and context-based interventions within the imaging process in reducing patient anxiety, increasing patient sense of control, and enhancing overall service satisfaction. These results provide evidence-based guidance for developing radiology workflows that prioritize the patient experience.

### 4.3 Current challenges and issues in implementation

The deployment of intelligent imaging triage systems faces several practical challenges. Firstly, in large healthcare facilities with multiple concurrent users or devices accessing the platform simultaneously, system stability may be compromised, potentially leading to performance bottlenecks and service disruptions, which could exacerbate patient anxiety. Secondly, AI systems may inherit biases from the training datasets. This may not be representative of the full demographic and disease spectrum, potentially resulting in suboptimal prioritization decisions, such as underestimating the needs of elderly patients. Thirdly, the integration of AI-driven platforms will require significant investment in educating current management staff on the algorithmic workings of the software and redefining clinical workflows. This may increase the overall workload and offset some of the anticipated automation benefits.

The digital divide presents a substantial obstacle for elderly patients, especially in China where individuals aged 60 and above increasingly constitute the imaging patient demographic. Many struggles with smartphone apps, find voice guidance challenging, or are unaware of QR code usage. These technological hurdles can result in missed intervention opportunities and increased anxiety. Existing literature on AI-based anxiety interventions is largely confined to single-center observational studies or short-term processes. No multicenter, randomized controlled trials with long-term follow-up have been published. Without such rigorous studies, assertions about the generalizability and sustainability of AI interventions remain speculative. Future multi-site, potentially international studies must adopt robust methodological designs to validate these systems’ efficacy and applicability across diverse patient groups and clinical contexts.

## 5 Emerging trends in imaging triage systems

### 5.1 Trends in technological development

Intelligent imaging classification systems are rapidly evolving, becoming increasingly complex, immersive, and standardized through integration with artificial intelligence in healthcare. These systems now extend beyond traditional scheduling to enhance patient experiences and address complex ethical, safety, and equity challenges. The foundational models have transitioned from rule-based approaches to predictive analytics, utilizing deep neural networks and mixed models that incorporate emotional status, physiological data, and patient clinical history to establish personalized prioritization frameworks. For example, the Mayo Clinic’s multimodal AI model analyzes cardiac function, physiological stress, and image quality from a single coronary angiogram, illustrating how AI and multimodal imaging can be fused to perform multiple analytical tasks in a single procedure (36).

Emerging immersive technologies, such as virtual reality (VR) and augmented reality (AR), hold promise for enhancing patient experiences by reducing wait times, alleviating pre-procedure anxiety, and aiding those with cognitive impairments. Controlled trials in the UK and Australia have demonstrated that patients exposed to VR-integrated imaging previews and emotional support content report significant anxiety reduction and improved satisfaction and information processing (37). These technologies can be explored in conjunction with 5G networks and brain-computer interfaces, with immersive systems integrated with AI-based scheduling to create seamless perception and feedback loops (38). However, the growing complexity of these scenarios necessitates ongoing attention to data security and ethical oversight. The development of AI-driven triage systems necessitates extensive medical datasets, which raises critical concerns regarding individual privacy, algorithmic bias, and transparency (38). Therefore, advancing explainable AI (XAI) methods, enhancing algorithmic transparency in academic publications, and conducting independent model evaluations have become important research priorities. Prominent institutions, such as Mayo Clinic and Stanford, advocate for ethical standards and transparency in model validation datasets, recommending guidelines for data visualization and imaging data content. They emphasize the ethical responsibility to address market incentives associated with compromised data records (39–42). The future of intelligent triage systems will go beyond “smart queue management,” exploring multi-purpose frameworks to map the spatial relationships between perceptual data, empathetic feedback, policy direction, and ethical governance (43).

### 5.2 Innovation trends in clinical application models

The integration of intelligent imaging triage systems in clinical practice is evolving from isolated applications to a comprehensive service-model overhaul (44). Future developments will go beyond optimizing queue efficiency to create an integrated “one-stop intelligent imaging service platform” that harmonizes workflow automation, routine emotional support, and personalized information exchange (45). Smart hospitals are incorporating AI-based triage systems into their imaging workflows, streamlining the process from registration to report acquisition (46). These systems efficiently manage resources and identify patient groups susceptible to anxiety, non-compliance, or language barriers. As a result, they initiate specialized pathways or emotional support, alleviating cognitive burdens and stress from complex procedures. A pilot study by the Mayo Clinic Health System reported that patient satisfaction with the waiting experience increased by 25% after implementing AI triage and patient behavior recognition technology, particularly among frequent visitors (27). Personalized queuing strategies have become critical for service models. By collecting real-time data on patient status, waiting behavior, psychological feedback, and responses to information prompts, the system dynamically adjusts queuing priorities and communication (47, 48). This patient-state-driven workflow model redefines “waiting” in healthcare, transforming it from a source of anxiety into an opportunity for service communication and emotional support (49–51).

### 5.3 Trends in interdisciplinary collaboration

Given the complexity and patient-centered nature of intelligent imaging triage systems, interdisciplinary collaboration is essential for their development (52, 53). In addition to radiology, disciplines such as psychology, nursing, information science, and management science must actively engage in the design, implementation, and ongoing enhancement of these systems (54–57). Psychologists play a critical role in developing strategies to assess anxiety, provide informational interventions, and evaluate semantic content to ensure that the system’s outputs effectively address emotions rather than merely conveying information (58, 59). Nurses, as frontline healthcare providers, contribute by documenting emotional responses influenced by contextual factors and linguistic nuances, which is critical for training AI models and enhancing their reliability and effectiveness (60–64). Furthermore, information systems engineers focus on addressing technical challenges, ensuring interface compatibility, maintaining technology, securing data, and sustaining system functionality to establish a robust clinical system. This collaborative effort is essential for the successful development and implementation of intelligent imaging triage systems in real-world healthcare settings. Interdisciplinary collaboration is essential for translating theoretical risk stratification models into practical workflows and tools (65, 66). This collaboration involves utilizing big data mining, machine learning to track data points, and monitoring clinical behavior changes to detect early signs of high-risk anxiety and non-compliance, which are associated with an increased risk of acute mental health issues (67). The collective capabilities of these organizations serve as a significant driver for change towards a patient-centered health system that emphasizes personalized care delivery and equitable health outcomes (68). The success of intelligent triage systems depend on the technologies employed and their ability to facilitate collaborative interdisciplinary efforts between the fields of medicine and engineering (69). This collaboration should integrate humanistic care principles with algorithmic development, recognizing the importance of teamwork across various health systems (70).

## 6 Future research priorities and recommendations

### 6.1 Critical issues requiring further exploration

Intelligent imaging triage systems offer potential in streamlining imaging workflows and alleviating patient anxiety stemming from wait times (71). Nonetheless, various unresolved obstacles impede the progression of this framework from proof-of-concept pilot studies to seamless integration into standard clinical practice. Existing risk stratification models are overly simplistic and lack the specificity required for personalized identification (72). Numerous studies oversimplify risk assessment by dividing patients into broad categories such as “urgent” or “non-urgent,” failing to consider the influence of additional unstructured variables (73–75). This constraint impedes the advancement of tailored triage approaches. Furthermore, current intervention systems primarily operate with one-way information transmission, lacking genuine intelligent interaction. Future research on anxiety relief is a key intervention target, prioritizing dynamic dialogue, personalized feedback mechanisms, and customized semantics to enhance human-machine collaboration and improve the patient waiting experience (76–78).

Recognizing these limitations, another challenge stems from our prevailing focus on immediate results, compounded by a broader deficiency in thorough, extended outcome evaluation. The lack of empirical data hinders our ability to address fundamental questions, such as the consistent impact of intelligent triage strategies on patient behavior or their effectiveness in improving healthcare resource utilization (79). These barriers underscore the vital need for multi-center, longitudinal data monitoring.

### 6.2 Recommendations for research design and methodology in future trials

To facilitate the clinical adoption and policy integration of intelligent imaging triage systems, it is imperative to transition from observational studies to a comprehensive, multi-stage research design that generates high-quality evidence. Although preliminary findings suggest potential benefits in reducing anxiety and enhancing patient satisfaction post-consultation, large-scale RCTs are urgently needed to validate these findings (80, 81). Prioritizing multicenter prospective randomized controlled trials in methodology will enhance external validity and assess the reliability and generalizability of the system across different hospital settings, regions, and patient cohorts (82). Implementing stratified randomization is essential to ensure comparable baseline characteristics among target patients and minimize the influence of confounding variables (83–85). Given the visibility of triage systems to participants actively engaged in the triage process, considering alternative blinding methods such as partial blinding and independent assessors is crucial to bolster the study’s rigor and credibility (86). Relying solely on quantitative metrics is insufficient for a comprehensive understanding of the factors influencing patient comprehension and their ability to take action (87). Hence, employing mixed-methods designs that integrate qualitative interviews with quantitative assessments is essential to gain deeper insights into patients’ behaviors, emotional responses, and decision-making processes. This integrated approach is particularly crucial for studies that demonstrate significant variations in anxiety reduction; qualitative data can elucidate potential intervention mechanisms and identify any shortcomings in the interventions. In addition to psychological assessment tools, physiological markers such as heart rate variability (HRV) and salivary cortisol levels can support findings in a cross-sectional manner (88, 89). Evaluating behaviors like appointment cancellations, follow-up visit frequencies, and the time between appointment requests and actual visits should be incorporated into efficacy assessments (90). Moreover, conducting cost-benefit analyses and evaluating medical resource utilization will establish a comprehensive validation process that encompasses various facets of intelligent therapy within the analytical framework.

### 6.3 Recommendations for policy and clinical utilization

The incorporation and utilization of intelligent imaging triage systems represent more than mere technological progress; they indicate an emerging restructuring of existing service delivery frameworks (91). To be successfully implemented, these systems must be closely linked with the aforementioned departments to jointly establish a support network involving all necessary institutions. At the policy level, integrating intelligent triage into national standards for constructing smart hospitals is crucial to offer policy backing for deploying these systems across different healthcare tiers and enabling initial pilot studies in hospitals. Special funds should be allocated for system development and platform maintenance, including effectiveness and dissemination studies, to facilitate the transition of this technology from controlled research environments to clinical practice (92). Reforming reimbursement mechanisms in medical insurance is essential for integrating AI-based triage services into reimbursement categories. Innovative payment models like bundled payment systems, covering the entire service pathway from emotional state screening to interactive feedback, can incentivize hospitals to seamlessly integrate intelligent systems into their billing processes. However, a key challenge is developing capacity among frontline healthcare staff. Training programs for imaging departments, IT departments, and nursing teams should prioritize system utilization, patient guidance, and early anomaly detection (93). Moreover, efforts to enhance skills should focus on monitoring human-machine interactions and identifying non-verbal emotional cues. To address the needs of elderly patients, multilingual and multi-platform educational interventions are crucial for enhancing their understanding, acceptance, and proficiency in system use, thereby reducing potential technological anxiety. By incorporating a patient-centered approach into institutional frameworks, intelligent imaging triage systems can transition from technical tools to catalysts for structural reform, advancing medical quality, efficiency, equity, and compassionate care.

## 7 Limitations of intelligent imaging triage systems

Although intelligent imaging triage systems show substantial potential in optimizing workflows, improving equipment utilization, and alleviating patient anxiety during waiting periods, several limitations hinder their widespread adoption. Firstly, clinical validation and long-term effectiveness of these systems remain insufficiently evidenced. Most existing studies focus on short-term outcomes and are primarily observational and single-center in nature, with limited large-scale, multi-center randomized controlled trials to provide robust evidence (7). Secondly, biases inherent in AI algorithms, particularly those resulting from unrepresentative training datasets, may lead to suboptimal prioritization, especially for underrepresented populations, such as elderly patients or those from low-resource settings (19, 20). Furthermore, the complexity of integrating these systems into existing hospital infrastructures presents a significant challenge, involving substantial upfront investments and extensive staff training. Lastly, concerns regarding data privacy and security are crucial, as these systems require handling sensitive patient data. Ensuring compliance with privacy regulations and safeguarding patient information are ongoing issues that must be addressed to facilitate broader implementation (94).

## 8 Conclusion

Intelligent imaging triage systems represent a promising application of artificial intelligence in medical imaging, with the potential to reduce waiting-related anxiety while improving workflow efficiency and patient experience. By optimizing scheduling, enhancing information transparency, and supporting patient engagement, these systems promote a shift toward patient-centered radiology services. However, current evidence is largely preliminary, relying mainly on single-center observational studies and limited outcome measures. Future research should prioritize rigorous multicenter trials, broader population representation, and comprehensive evaluation frameworks. Addressing challenges related to data privacy, equity, and digital accessibility will be essential for the safe and effective integration of intelligent imaging triage systems into clinical practice.
